# An ethical comparison of living kidney donation and surrogacy: understanding the relational dimension

**DOI:** 10.1186/s13010-019-0080-9

**Published:** 2019-09-18

**Authors:** Katharina Beier, Sabine Wöhlke

**Affiliations:** Department of Medical Ethics and History of Medicine, University Medical Center, 37073 Göttingen, Germany

**Keywords:** Family, Personal relationships, Altruism, Motherhood, Reciprocity, Commercialization, Third-party assisted reproduction, Comparison

## Abstract

**Background:**

The bioethical debates concerning living donation and surrogacy revolve around similar ethical questions and moral concepts. Nevertheless, the ethical discourses in both fields grew largely isolated from each other.

**Methods:**

Based on a review of ethical, sociological and anthropological research this paper aims to link the ethical discourses on living kidney donation and surrogacy by providing a comparative analysis of the two practices’ relational dimension with regard to three aspects, i.e. the normative role of relational dynamics, social norms and gender roles, and reciprocity. Based on this analysis, we derive conclusions for the framing of living organ donation and surrogacy in ethical theory and practice.

**Results:**

First, our analysis emphasizes the relevance of acknowledging the complex relational implications of living kidney donation and surrogacy. Underestimating this relational dimension may not only lead to individual crises but endanger existing as well as newly emerging familial relationships. Second, we point out differences in the normative assessment of social norms and gender roles in the ethical debates about living kidney donation and surrogacy. In particular, we show how different evaluations of altruism affect the understanding of autonomy in both contexts. In addition, we sensitize for biased perceptions of gender roles. Finally, we argue that challenges resulting from unresolved reciprocity are an issue in living kidney donation and surrogacy independent of whether the exchange of body parts or bodily services is framed as a gift or commercial exchange. By pointing out the limits of financial compensation, we stress the relevance of non-material, relational rewards as potential remedy.

## Introduction

The use of body parts or bodily functions for the sake of others is an ethically contested field. In particular, when organs or bodily functions of living persons are involved, as for example in living organ donation or surrogacy, this raises fundamental ethical questions. Living donation implies the transplantation of an organ or parts of it from a living person to a patient suffering from, for example, end-stage renal failure or different liver diseases. Surrogacy in its most general definition means that a woman (surrogate mother) bears a child for another couple or single person. Although the practices of living organ donation and surrogacy differ from each other in several respects, it is striking that the bioethical discourses revolve around similar ethical questions and moral concepts. For examples, challenges with regard to autonomy [1, 2], commercialization [3–6] or exploitation [7–10] are taken up for both practices. Despite these convergent challenges, the ethical discourses on living donation and surrogacy grew largely isolated from each other. If at all, comparisons between the two debates are cursorily drawn and with strategic intentions, for example, in order to justify the commercialization of organ donation [11] or, vice versa, to promote an altruistic framing of surrogacy [12].

Against this background, we aim to link the ethical discourses on living donation and surrogacy. The central anchor point for our comparative analysis is that both are genuinely relational practices. However, the ethical consequences of this relational dimension are still underexplored. This is not to suggest that living donation and surrogacy are identical practices; rather our goal is to highlight existing *parallels and divergences* with regard to the normative role and ethical implications of personal relationships in both fields. In particular, we provide a comparative analysis of the two practices’ relational dimension with regard to three aspects and derive some conclusions for the framing of these practices in ethical theory and practice. Methodologically, our analysis is based on a broad review of ethical, sociological and anthropological research on living donation and surrogacy.

In the following, we will direct our focus primarily on living kidney donation (LKD) within personal relationships for two reasons.^1^ First, kidneys are the most common organ transplants because kidneys represent the most common organ failure. At the same time, LKD is of comparatively low risk. Second, many countries restrict LKD to relatives or close friends due to high tissue compatibility (HLA matching between family members) and in order to avoid organ trafficking [15]. With regard to surrogacy, we restrict our analysis to commercial gestational^2^ surrogacy amongst (former) strangers as this is globally seen the most common practice. While regulative frameworks distinguish between compensation for the expenses of surrogacy and genuine payments to surrogates, it is important to note that there is a thin line between the two types [16]. In our analysis of surrogacy, we thus refer to empirical results from both contexts.

For the comparison of LKD and surrogacy we introduce three aspects (section 2). We first map out specific relational dynamics in both fields and discuss normative and practical implications (section 2.1). Second, we analyze the role of social norms, gender stereotypes and body concepts that underlie current practices and related ethical discourses on LKD and surrogacy (section 2.2). Third, we outline specific challenges that arise from (unresolved) reciprocity in both fields and discuss potential remedies (section 2.3). Finally, we draw some conclusions regarding the ethical and practical framing of LKD and surrogacy (section 3).

## Comparative analysis

### The moral relevance of familial relationships and dynamics

In ethical debates on LKD relational aspects did not play much of a role for the reason that protection of donors was regarded the most important issue for a long time [17–19]. Specifically, close relationships as a complex, interpersonal context from which decisions about living donation arise remained unconsidered [20]. In fact, donors and recipients used to be portrayed as individuals who act and decide as independent, autonomous agents. This perception is still reflected by current procedural requirements. For example, the German Transplantation law requires that each living donation is reviewed by a specially appointed commission.^3^ Its primary task is to guarantee the voluntariness of donation and exclude trading in organs. Due to this focus, the process of decision-making, particularly with regard to the family dynamics involved, has long remained a black box and long-term data on the development of familial relationships after living donation are still missing.

As studies have shown, even living organ donors and recipients themselves do not necessarily recognize the relevance of intra-familial decision-making dynamics and their implications. For example, when German kidney donors and recipients were asked about the voluntariness of their decision, they adopted the viewpoint of the legal framework, which treats the prevention of organ trafficking as the only voluntariness impairing factor. On the one hand, donors and recipients expressed their trust that the legally required inspection process is effective in excluding illegal practices such as organ trade. On the other hand, they also pointed to specific limitations of this inspection by stressing that voluntariness within familial decision-making is difficult to verify. In fact, intra-familial donation can hardly be questioned or rejected in cases where both, donor and recipient, express their willingness [13]. In this regard, the inspection process by the commission is deemed problematic by organ donors and recipients [18, 21].

In recent studies, there is raising awareness for the complexities of familial decision-making on living donation [21, 22]. Familial decisions, by nature, are determined by relational concerns that are not adequately captured in terms of individual autonomy and voluntariness [23, 24]. Most strikingly, family members’ moral values impact the decision-making process. This can set off specific intra-familial dynamics in the run-up to living donation. For example, given their willingness to donate an organ, donors often take on a dominant role in the decision process. Also, by framing their donation as a gift, donors ascribe the latter a high moral value which makes it difficult for recipients to reject their offer [20, 25].

The relationship of the kidney donor and the recipient is influenced by concerns about the donated organ’s maintenance after transplantation. Such concerns include the donor’s expectation that the recipient will handle the organ with care in order to ensure that it will not be rejected by the recipient’s body. It is important to note, however, that LKD impacts familial relationships in different ways. Parent-child relationships, for example, tend to become more intimate [18, 19], whereas sibling pairs or married couples are more likely to face conflicts in their relationships, for example due to controlling or autonomy-restricting behavior [26]. Amongst siblings, for example, a simple greeting gesture, where the donor asks the recipient “how are you?” has been shown to be interpreted by the recipient as a kind of control, whether he lives a health-conscious life with the donated kidney [27]. In general, there is evidence that the bodily exchange of organs leads to an intensification of preexisting relational dynamics amongst family members – for better or worse [17, 28]. Moreover, family ideals may even imply an obligation to organ donation for specific family members. Once again, this emphasizes the complex nature of intra-familial decision-making – a fact which, amongst others, requires more nuanced perceptions of voluntariness in LKD [29].

Surrogacy, by nature, has relational implications that spill over the legal barriers of contracts [30] and “(a)rm-lenght liberal market transactions” [31]. In particular, during pregnancy, the surrogate mother inevitably bonds with the child. In turn, the child’s “narrative of existence” [32] is irreversibly linked to the surrogate’s contribution. In a study conducted by Zadeh et al. [33] the majority of adolescents wished to get to know their surrogate mother and to be in contact with her. However, due to the still pervasive ideal of the genetically based nuclear family and the wish for an unequivocal assignment of motherhood/parenthood [31] surrogate mothers and intended parents are often perceived as distinct (legal) parties whose interests must be secured one against the other [34]. In practice the surrogate is then removed from the “scripts of family intimacy” [35] and her special relationship with the child neglected [36]. As studies have shown, however, for rendering surrogacy a satisfying experience for all parties, including the surrogate, the existence of an ongoing relationship beyond the birth of the child is important [37, 38]. In fact, if there is room for an ongoing contact between surrogates and children this may even include the surrogate’s own children and grandchildren [33].

Surrogacy also creates a relationship between the child and the intended parents. Merely regarding them as commissioners seems inconclusive from the child’s point of view. The child is not only “mentally conceived” by them [10]; but, at least in gestational surrogacy, the intended parents also make bodily contributions to the child’s existence. Thus, given their “morally transformative causal role” [10] in the child’s creation, they are more than just gamete donors [39]. However, if surrogacy is practiced in countries that assign motherhood to the woman who gives birth, the intended parents’ relationship with the child is likely to remain unacknowledged.

Relational dynamics do also play a role in surrogacy. In particular, the surrogate mother, by promising to carry a child for another person or couple, and the intended parents, by requesting and/or accepting this outstanding bodily contribution, inevitably enter into a relationship [36, 40, 41]. At least, studies from the UK and USA show that the decision for starting with surrogacy is only taken after prior contact and the existence of mutual sympathy [42, 43].

However, relational challenges may arise from surrogacy, too. For example, the potential “annexation” of the other’s body/life that has been described for LKD can also occur in surrogacy. While in LKD, it is the donor who might be tempted to interfere with the recipient’s life in surrogacy it is rather the “donor”, i.e. the surrogate mother, who is likely to face control by the recipients, i.e. the intended parents. Specifically, their wish to take part in the pregnancy (e.g. by accompanying the surrogate to check-ups and ultra-scans) may be experienced by the surrogate as intrusion into her private sphere. On the other hand, it has been shown that surrogate mothers have an interest in continuing the relationship with the intended parents, and particularly the intended mother, even after surrogacy [40]. Therefore, while living organ donors face the challenge of “letting the other go”, actors in surrogacy need to be prepared to “letting the other coming closer” and to handle the complex familial relationships that this practice entails.

Consequently, there is a need to prepare actors involved in LKD and surrogacy arrangements for these relational challenges, e.g. by counselling. Insofar voluntariness within families is difficult to verify, the initiation of an open dialogue about the relational dimension of decision-making may help kidney donors and recipients to cope with this experience. In particular, the determination of a particular family member as donor which, at first sight, might appear without alternatives, could be questioned during counselling and in this way broaden the horizon for familial decision-making and voluntariness. In surrogacy, it is important to discuss the mutual expectations of surrogate mothers and intended parents, particularly with regard to future familial involvement and contact [36]. Moreover, counselling should not only include all parties involved but also be offered as a *process* that lasts also after the time of transplantation or the birth of a child respectively. Although psychosocial evaluation in the run up to LKD is widely established and awareness for the dynamics of recipient donor-relationships exists [44], the major focus is still on the assessment of donors rather than the mutual dynamics between donors and recipients [45, 46]. Consequently, there is need to include this aspect in a more systematic manner in the psychosocial evaluation process that precedes living donation. In the context of surrogacy, there is increasing awareness of the relevance of counselling. For example, in the UK the Human Fertilization and Embryology Authority requires in its Code of Practice [47] that “implications counselling” should be offered to all parties involved in a surrogacy arrangement. The Ethics Committee of the American Society for Reproductive Medicine recommends counselling as “an adjunct to the legal agreement to help each participant understand and communicate his or her needs and/or expectations” [48]. Moreover, also for surrogacy in countries like India there are calls for “in-depth counseling of all parties engaged in surrogacy arrangements” [49]. In practice, however, counselling is still limited and opaque. For example, it is often up to the clinics if, to what extent, and to whom counselling is offered. Particularly where surrogacy is left to the rules of the market, counselling boils down to the exhortation of surrogates to fulfill their contractual duties. In this setting no space seems to exist for the taking up of a forward-looking perspective that involves mutual concerns of intended parents *and* surrogate mothers. Legal bans on surrogacy display an additional barrier to the provision of adequate counselling. In Germany for example, the criminalization of professional assistance to surrogacy also includes the provision of psychosocial counselling. Thus, if German intended parents make use of surrogacy abroad there is a risk that not only surrogates but also they themselves are insufficiently prepared for the relational complexities of this practice.

### Social norms, gendered roles, and body concepts

The medical option of LKD has developed a momentum of its own. The expectation to donate an organ is no longer seen as an individualized act but a matter of societal solidarity. In the public discourse the focus is on: “How can more organs be transplanted?” [50]. In a similar vein, LKD is predominantly depicted as a positive action in public and medial representations. Thereby the donated organ is increasingly perceived as something that is endowed to one’s next of kin in a medical emergency or even something one should give [27]. In the context of intra-familial donation, notions of mutual obligation take on a specific shape against the background of established roles within the family [51]. In fact, studies focusing on parents who donated an organ to their children reveal that not all potential familial options for donation were discussed within the family insofar as mothers often regard themselves as the only option for securing the survival of their child [17, 26]. Especially women with a traditional understanding of gender roles regard LKD as an expansion of their familial obligations [27, 29]. This is not least reflected by the fact, that two-thirds of the kidneys donated every year stem from women [52].^4^

Social expectations for donation are to a large extent also constituted by a reference to gendered body concepts. As it has been shown, women tend to allude to holistic body concepts. In particular, they stress the reproductive capacity of their bodies by drawing direct comparisons between birth, motherhood and the mother-child relationship [27]. In this regard, the familiar image of the self-sacrificing mother functions as a cultural mechanism that enables the transplant endeavor [55]. However, a mother’s decision to become a donor is neither to be viewed as “yet another means of gender exploitation” [55] (ibid., 7) nor as an infringement of her autonomy. Specifically, Crowley-Matoka and Hamdy point out that “women who defined themselves first and foremost as mothers often expressed pure elation and relief on news that they could donate their kidneys to their sick children” [55] (ibid.). The fact that mothers can benefit from donation themselves, for example, by experiencing high social recognition and prestige within their social setting, displays another argument challenging the notion of exploitation [56]. As regards the autonomy of mothers but also other family members, the latter’s decision to donate an organ is often framed as a matter of relational autonomy [27, 57]. As it has been noted “to be autonomous, in a relational sense, is to be responsive and responsible to others, and interdependent within complex networks of relationships, which will not always easily accord with the practices and expectations we have normalized in cultures that have elevated ‘the individual’” [58]. From this perspective then, love, affection and concern with others’ needs are not seen as limiting autonomy but as enabling it [17, 59]. Still, there is no inevitability of mothers becoming donors to their children. By referring to a French study which revealed that the medical staff were more likely to urge fathers to serve as living donors because “mothers have already done their part,” Gauthier stresses the availability of alternative conceptions of bodily responsibility [60].

As an exclusively female contribution, also surrogacy involves specific social and gender norms. However, their evaluation differs from the context of LKD. In particular, the notion of altruism appears at least ambivalent. On the one hand, in contrast to commercial surrogacy, surrogacy following from altruistic motivation is seen as a socially and ethically acceptable act. This appreciation of altruism, however, comes with a price, particularly for surrogates in developing countries. Specifically, commercial agencies take strategic advantage of the socially accepted norm of altruism by emphasizing the wish of surrogates to help a childless couple and downplaying the commercial dimension. As a consequence, this restricts the options for surrogates to negotiate about their payment as the cultural norm of gift-giving leaves the definition of a counter-gift to the donee [61]. On the other hand, the notion of ‘the selfless women’ is far more critically scrutinized than in the context of LKD. In particular, altruism in surrogacy is problematized as a stereotyped gender norm which assumes a natural disposition of women to give [62]. In this regard, altruistic arrangements are criticized to be at least as exploitive as commercial ones [63]. Following from this is a general suspicion about surrogates’ autonomy [64]. Specifically, surrogates are suspected to act out of “false consciousness”, for example, due to feelings of guilt or concern with others’ needs, which would restrict their autonomy [65, 66]. Although it has been shown that surrogates, at least at the individual level, do also take a sense of pride and self-fulfilment from their action [67], in contrast to LKD there is no comparable social recognition for them. Rather surrogates tend to be discredited as “bad mothers” who give up their children. Obviously, the social norm of being a “good” mother, involves a double-bind in the context of surrogacy. Specifically, if a woman takes on the task of surrogacy in order to provide a living for her children – something that is usually associated with being a good mother – this requires her at the same time to relinquish the surrogacy baby. Lack of social support or even social discrimination and stigmatization, however, render surrogate mothers additionally vulnerable [68].

Since LKD and surrogacy involve the crossing of body boundaries, body concepts play a role in both contexts, though with different accentuation. Kidney donors often invoke holistic conceptions of the body [27]. The latter involve the idea that there exists a connection between a person’s identity and his organs. In surrogacy, in contrast, the “provided” body part, i.e. the womb, tends to be separated from the rest of the body. This bodily fragmentation is not only a strategy enforced by surrogacy agencies in order to ensure the child’s relinquishment [69] but surrogates internalize this perception for themselves in order to leave their identity as mothers to their own children intact [40]. Recent research has shown, however, that this fragmented perception of the body is not a given in surrogacy. By using the metaphor of the “shifting body” Teman describes the “process of disjoining pregnant embodiment from the surrogate’s embodied self and rejoining it to the embodied experience of the intended mother” [38]. According to this interpretation, the realization of the two women’s individual agendas depends on their relationship with one another. “The surrogate cannot disembody the pregnancy and distance the fetus without the intended mother’s reciprocal containment and support” [38]. As Teman argues, the concept of the “shifting body” could be “useful in thinking about living donors’ interactions with recipients of their organs”. In fact, this is also a process “which requires each side to undergo redefinitions of the links between body and self” [38].

### The challenge of unresolved reciprocity

The ethical framing and practice of LKD is strongly influenced by the ideal of selfless, altruistic giving in contrast to commercialized practices. Yet, it has to be noted that gifts, as a cultural rule, are never unconditional but inevitably establish an obligation of the recipient towards the donor. Fox and Swazey (1992) have prominently illustrated how difficult and desperate this obligation is already for the recipient of a post-mortem organ and described this phenomenon as the “tyranny of the gift” [70]. The cultural rule of providing a return gift as an expression of gratitude is even more present and, at the same time, more invisible in living donation. In fact, the concept of donation which emphasizes the desired norm of altruism denotes, in its basic form, a one-sided act. However, in LKD this obscures the fact that the decision for donating a kidney is embedded in a complex web of reciprocal dynamics [27, 59, 70]. However, donors and recipients are also not necessarily aware of the implications of reciprocity within the private realm of the family. Reciprocal dynamics that are spurred by feelings of gratitude, especially after transplantation, can thus lead to irritation in those affected [71]. In particular, the desire for reciprocation amongst recipients remains often unfulfilled. Recipients report occasionally that they wish to be able to counteract the enormous social pressure spurred by the act of donation through a financial compensation [21]. While payments are legally excluded for LKD in most countries, there is discussion whether a “rewarded gift”, i.e. giving financial compensation to donors, would be helpful to relieve recipients from potentially problematic dynamics, such as, unresolved reciprocity, guilt or social pressure, within the family [6, 72].

In this regard, debates about compensation might take a new direction insofar as  compensation as a means to restore symmetry in family relationships can be morally distinguished from compensation as an incentive to increase the number of donors [27, 73]. Specifically, in light of the former motive, payments would be a means to handle intra-familial tensions that arise from unresolved reciprocity rather than a sign for morally dubious commercialization [74–76]. At the same time, it is important to note that the prominent dichotomies of gift vs. commodity and altruism vs. market are inadequate to reflect the complex relational motivations that are present in living donation [6, 77]. Various studies on LKD pointed out how people divest parts of their bodies and convert them into economic goods [4, 73, 78]. In this regard, the gift is no longer the epitome of altruistic action, just as little as commodities only stand for purposive action [72].

The complex role of reciprocity is also obscured in surrogacy. Specifically, the exchange of reproductive services for money promotes the impression that the surrogate and intended parents are even with each other. According to this understanding, the surrogate mother does not have any further task than gestating the child and the intended parents do not owe her anything beyond the money. This perception is illustrated by an intended parent’s saying that “the SM [surrogate mother] had been paid for all her services, and there was no need to feel indebted or contact her further” [79]. However, although commercial surrogacy entails a form of mutual exchange, this may not suffice to avoid the challenge of unresolved reciprocation. While in LKD this is mostly an issue at the recipient’s side, in surrogacy problems of unresolved reciprocity rather arise at the donor’s, i.e. the surrogate mother’s side. Specifically, by referring to their action as a “gift” surrogates emphasize that compensation “may be insufficient to erase certain relationships, and that the relational element may continue to survive despite the monetary exchange” [80].^5^ In this way they do not only acquire a moral argument for maintaining an ongoing relationship with the newly created family, but also counter the stigma of “baby-selling” [61]. At the same time however, surrogate’s expectation of lasting indebtedness, invoked by the gift metaphor can conflict with the intended parents’ perception of surrogacy as an outright commercial transaction [81, 82]. In particular, they may not share the understanding of surrogacy as transcendental gift that comes along with a more far-reaching sense of reciprocation [83].

While in LKD, financial compensation is discussed as means to increase the recipient’s agency by giving the donor something in return, compensation in surrogacy serves as a means to account for the giver’s, i.e. the surrogate’s contribution. However, in most legislation, payment for the provision of body parts or bodily services is subject to moral criticism and thus also legally barred. When looking from the perspective of relationships, however, the role of monetary exchange requires further scrutiny. For example, payments in surrogacy may allow for more symmetric relationships by freeing women from the expectation of being “natural givers”. Moreover, it has been argued that “given the enormity of [surrogate mothers] gift, it would be wrong for the intending parents not to reciprocate by giving something substantial in return” [83]. The question is, however, whether “something substantial in return” can ever be sufficiently expressed in economic terms alone. At least from a moral point of view, it seems that reciprocation would have to go beyond this by acknowledging the surrogate mother’s special role in the arrangement and particularly her relationship towards the child [31, 36, 84]. As people are part of complex social networks which entail both, aspects of intimacy and economy [82] it may be even obstructive to perceive of surrogacy as an either altruistic *or* commercial act. For this reason, compensating surrogate mothers for their efforts may not per se expel other-regarding motivations such as the wish to help another couple [82, 85].

## Conclusions

Our comparative analysis revealed several overlaps with regard to the relational implications of LKD and surrogacy. In particular, from our analysis the following conclusions can be drawn.

First, the complex relational implications of LKD and surrogacy need to be acknowledged. Underestimating this relational dimension – either by taking relationships as unwavering basis like in LKD or by disguising them as in the context of commercial surrogacy – may not only lead to individual crises but endanger existing as well as newly emerging familial relationships. Irrespective of whether bodily exchange practices occur between intimates or strangers, it is not only voluntariness that matters, but also the stability of relationships as well as their adjustability to new situations. Given that LKD and surrogacy have far-reaching relational implications decisions need to be well-considered beforehand. This seems particularly relevant in light of recent developments in living uterus transplantation [86]. Given that donors are typically family members or close friends, the inherent relational dimension of donation, which is reinforced by the very nature of reproduction, should not be underestimated. Therefore, in order to withstand relational tensions in LKD and surrogacy an ongoing and stable relationship as well as clear communication between all parties involved is advised. This provides an argument in favor of professional psycho-social counselling of donors and recipients rather than one-sided access control. However, it is advisable to provide counselling in LKD and surrogacy with different emphasis. In LKD it is important to prepare donors and recipients for handling potentially burdensome feelings of guilt as well as expectations for indebtedness within their relationship. In surrogacy, it is specifically important to raise awareness that engaging another woman for child-bearing by nature creates ongoing relationships and responsibilities which cannot be invalidated by the intended parent’s payment for the surrogate mother.

Second, our comparison of social norms and gender roles in the ethical debates about LKD and surrogacy reveals differences in the normative assessment of altruism which impact the understanding of autonomy in both contexts. In debates on LKD, giving an organ is not only regarded reconcilable with autonomy but almost seen as a natural act amongst family members, particularly mothers. Debates on surrogacy, in contrast, invoke suspicion about autonomy-diminishing influences even if it is practiced amongst intimates. In addition, bearing a child for others is problematized in terms of women’s physical and psychological fragmentation that may be to the detriment of children and thus contradict the norm of being a good mother. However, this “black and white” perception of surrogacy may be ill-advised. In order to avoid the condemnation of surrogate mothers and stigmatization of children born from surrogacy it is important to get a more nuanced picture of the motivations involved. In this context it would be also important to critically assess whether these different evaluations of altruism and women’s agency in LKD and surrogacy are morally justified.

Third, from our juxtaposition of prevalent social and gender norms in LKD and surrogacy, two further insights can be derived. For one thing, the problematization of implicit assumptions about women’s natural disposition to give in the context of surrogacy helps raising awareness that there is no such disposition for women in LKD either. As living donation, in principle, can be accomplished by men and women, there is reason to encourage more gender equality in donation. For another thing, the comparison of LKD and surrogacy urges us to rethink our perception of the latter as a per se fragmentizing experience. While LKD has been described as an experience that may both, strengthen and endanger relationships, there is no reason to expect only detrimental relational effects from surrogacy. In fact, more recent analyses of surrogacy as an embodied relationship [87] call for more nuanced perceptions of surrogacy, too.

Finally, our analysis reveals that reciprocity, and particularly struggles that result from unresolved reciprocity is an issue independent of whether the exchange of body parts or services is framed as a gift or commercial exchange. Consequently, in the run-up to LKD and surrogacy, it is important to address the moral implications of reciprocity. Furthermore, the results of our comparative analysis contribute to the ongoing discussion about remuneration in living donation. While this might be effective in relieving recipients from the “tyranny of gift”, it is not clear whether this would be equally satisfactory for donors. At least, there is evidence from the context of surrogacy that surrogate mothers, who take on the role of a donor in this context, still expect an act of reciprocation beyond financial rewards. Thus, material compensation may not suffice to solve the issue of unresolved reciprocity. As there is no predetermined counter-gift, neither for donating an organ nor for bearing a child for others, we are thrown back to the relationship itself. However, non-material forms of reciprocation are still not sufficiently established. While reciprocity in LKD is at least handled at an individual level, in surrogacy reciprocation beyond monetary compensation is hardly discussed. A change to this situation would require acknowledging the complex familial relationships that arise from surrogacy and provide for an acceptable role for all parties involved.

## Data Availability

All data generated and analysed during this study are included in the published article.

## Abbreviations

HLA: Human Leukocyte Antigens
LKD: living kidney donation

## Footnotes

1 While the majority of countries restrict LKD to personal relationships, including next-of-kin, but also friends, (e.g. Germany, The Netherlands) [13], there are also countries that accept donation amongst unrelated donors (e.g. Iran, Philippines) [7]. A further differentiation exists regarding whether living organs can be directly donated to a specific recipient or indirectly to a pool of organs from which they are distributed according to predefined criteria (e.g. Unites States) [14].

2 Gestational surrogacy implies that the surrogate mother has no genetic link with the child; the gametes are either derived from the intended parents or gamete donors.

3
https://www.dso.de/homepage-english

4 This gender difference in donation rates is not a German phenomenon only but can be observed in all countries with altruistic (i.e. non-commercial) frameworks in place [25, 53, 54].

5 Intended parents use the gift-metaphor particularly in the context of traditional surrogacy. Ragoné explains this by the importance of genetic ties that constitute kin relationships – an aspect that, according to prevailing cultural norms, is more difficult to deny [69].
